# *Pelecyphora chihuahuensis* (Britton & Rose) D. Aquino & Dan. Sánchez: A Review on Its Taxonomy, Ecology and Conservation of an Endemic Mexican Cactus Species with Biotechnological Perspectives

**DOI:** 10.3390/biology15050413

**Published:** 2026-03-03

**Authors:** Fernando Daniel Loweree-Rivera, Sandra Pérez-Álvarez, Alicia Melgoza Castillo, José Humberto Vega Mares, Héctor Alejandro Leyva-Hernández, Esteban Sánchez Chávez, César Marcial Escobedo-Bonilla, Luisa Patricia Uranga-Valencia, Jesús Alicia Chávez Medina

**Affiliations:** 1 Facultad de Zootecnia y Ecología, Universidad Autónoma de Chihuahua (UACH), Perif. Francisco R. Almada Km 1, Pavis Borunda, Chihuahua C.P. 31453, Mexico; fernando.loweree@gmail.com (F.D.L.-R.); amelgoza@uach.mx (A.M.C.); jhvega@uach.mx (J.H.V.M.); 2 Facultad de Ciencias Agrícolas y Forestales, Universidad Autónoma de Chihuahua (UACH), Km 2.5 Carretera a Rosales, Delicias C.P. 33000, Chihuahua, Mexico; luranga@uach.mx; 3 Unidad Regional Los Mochis, Universidad Autónoma de Occidente, Blvd. Macario Gaxiola, Los Mochis C.P. 81217, Sinaloa, Mexico; hector.leyva@uadeo.mx; 4 Centro de Investigación en Alimentación y Desarrollo AC, Unidad Delicias, Cd., Delicias C.P. 33089, Chihuahua, Mexico; esteban@ciad.mx; 5 Instituto Politécnico Nacional-CIIDIR Unidad Sinaloa, Juan de Dios Bátiz Paredes No. 250, Guasave C.P. 81101, Sinaloa, Mexico; cescobe@ipn.mx (C.M.E.-B.); aliciachavezm@hotmail.com (J.A.C.M.)

**Keywords:** cryopreservation, endemism, habitat fragmentation, micropropagation, phylogenetics

## Abstract

The cactus *Pelecyphora chihuahuensis* is endemic to northern Mexico and represents an interesting subject on the integration of classical taxonomy with modern biotechnological tools to solve conservation issues. Because of its narrow ecological range and high ornamental value, the species is increasingly at risk from degradation of its habitats, climate change, and plant poaching. This review includes current knowledge on its taxonomic status, ecological distribution, and conservation needs, with a focus on biotechnological means to aid its preservation. Aspects such as molecular markers, next-generation sequencing, and previously reported GIS-based species distribution models provide valuable insights into its identity and ecological niche. Biotechnological tools for *ex situ* conservation include *in vitro* propagation and cryopreservation. Potential applications of CRISPR-Cas and synthetic biology in preserving rare cacti are also discussed. By uncovering gaps, this review opens a window of opportunity to urgently promote the sustainable management of *P. chihuahuensis* and related endangered cacti by merging biotechnology with ecology and taxonomy, the results presented here underscore the importance of integrating scientific findings into future research that supports the development and implementation of effective policies that prioritize the conservation and biocultural preservation of arid-land flora, ensuring that both ecological integrity and cultural values are maintained for these unique ecosystems.

## Simple Summary

The cactus *Pelecyphora chihuahuensis* is endemic to northern Mexico. It has become a threatened species due to its secluded habitat, climate change and poaching. The latter makes it a highly vulnerable species due to its restricted distribution and high ornamental value. This review delineates the taxonomic position of the species, its ecological range, and its urgent conservation needs, along with the use of biotechnological tools as the main conservation solution. Molecular markers coupled with next-generation sequencing and species distribution models based on GIS give insight into its identity and ecology. Structures such as *in vitro* propagation and cryopreservation could offer feasible approaches for *ex situ* conservation of the species, with CRISPR-Cas and synthetic biology offering conservation possibilities in the future. Integration of taxonomy, ecology, and biotechnology will provide a basis for sustainable conservation and policy development.

## 1. Introduction

The family Cactaceae is a fundamental part of arid and semi-arid ecosystems across the Americas, with Mexico serving as its top center of diversity [1]. Beyond their ecological roles, cacti hold deep cultural and economic value, especially within Mexican biocultural heritage [2]. The Chihuahua Desert, a key ecoregion spanning Mexico and the United States, is recognized as a global hotspot for cactus diversification and endemism [3]. This unique concentration of specialized flora faces increasing human and environmental pressures, making the study and conservation of its endemic species a priority [4,5].

*Pelecyphora chihuahuensis* (Britton & Rose) D. Aquino & Dan. Sánchez is a small, globose cactus highly valued for its ornamental appeal, which unfortunately makes it a target for illegal collection. Morphologically, the species is distinguished by its reduced dimensions and intricate spination, reflecting evolutionary adaptations to the xeric environments of the Chihuahuan Desert. While traditionally described as endemic to the central region of Chihuahua, specifically in microphyllous and rosetophyllous scrublands [6,7], its fundamental niche covers approximately 14,484 km^2^ within an altitudinal gradient of 1217 to 1574 m [8]. The species thrives in arid to semi-arid conditions (BWhw and BShw), characterized by mean annual temperatures between 16.5 and 19.3 °C and low annual precipitation ranging from 288 to 411 mm [8]. Despite these ecological characterizations, reports of its presence extending into northern Durango [5] suggest that its actual geographical limits remain partially unresolved. This spatial uncertainty underscores the necessity for updated ground-truthing and high-resolution niche modeling to refine its conservation status and guide management efforts.

The taxonomic history of *P. chihuahuensis* highlights the difficulties in cactus systematics. Described initially as *Escobaria chihuahuensis* [9], the species embarked on a long taxonomic journey. Over the next century, it was reclassified and moved to other genera [10,11], reflecting changing interpretations of morphological features within the mammilloid complex. This taxonomic instability is not just academic; it complicates conservation efforts, obscures legal protection, and hampers accurate threat assessments. It is only through molecular phylogenetics that this long-standing uncertainty has been clarified. A comprehensive study by Sánchez et al. [12] provided substantial evidence to transfer the species to the genus *Pelecyphora*. This reclassification demonstrates how modern biotechnological tools are essential for resolving complex taxonomic issues, thereby establishing a stable foundation for conservation strategies.

Despite its evolutionary adaptations, *P. chihuahuensis* faces severe anthropogenic threats. The biggest one comes from illegal poaching, driven by the high ornamental value of rare cacti in the international market, a risk shared by many Chihuahuan Desert species [13]. At the same time, its specialized habitat is becoming increasingly vulnerable to land-use changes. Converting desert scrubland for agriculture and extensive cattle grazing are significant causes of habitat loss and fragmentation throughout the region [4]. In addition to these immediate threats, the global challenge of climate change poses serious risks to endemic species with narrow ranges by potentially shifting their environmental niches beyond their capacity to adapt [8]. The combined effects of these threats place *P. chihuahuensis* in a fragile position, requiring urgent conservation efforts.

Tackling the interconnected issues of taxonomic confusion, ecological uncertainty, and severe human impacts requires a comprehensive scientific approach. This review contends that modern biotechnology provides a valuable toolkit to support traditional conservation methods for endangered cacti. Here, we compile current knowledge on the taxonomy, conservation, public policy and conservation status of *P. chihuahuensis*, with a focus on the potential of biotechnological solutions. Specifically, authors explore how molecular tools can help clarify its taxonomy, how *in vitro* micropropagation and cryopreservation can preserve genetic resources outside their natural environment. By integrating these fields, this review aims to establish a scientific foundation for developing robust, evidence-based strategies and policies to ensure the survival of this Chihuahuan Desert cactus and other threatened plant species.

## 2. Systematic Background

The taxonomic history of *P. chihuahuensis* shows the evolution of systematic standards within Cactaceae, from traditional descriptive morphology to modern genomic methods approaches. The important timeline of its taxonomic classifications is outlined below.

### 2.1. The Morphological Era: The Escobaria vs. Coryphantha Conflict (1923–2005)

**Figure 1:**
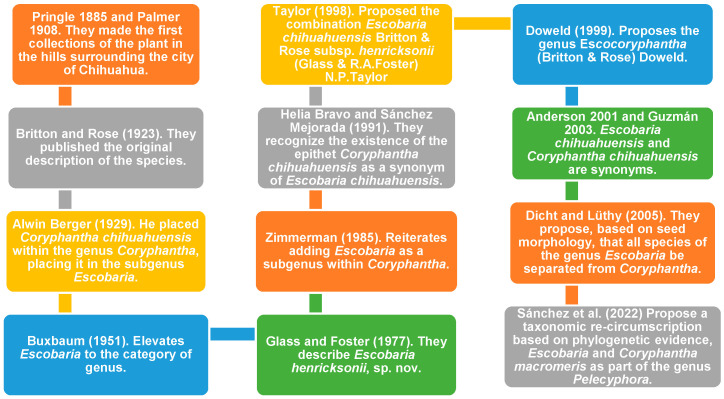
Historical taxonomic timeline of *Pelecyphora chihuahuensis*. The diagram illustrates the major generic re-classifications and nomenclatural changes, tracing the species from its initial collection to its current phylogenetic placement within *Pelecyphora sensu* Sánchez et al. [12]. Note: Names and years within the diagram represent historical milestones and botanical collectors; the scientific support for these events is provided through the numbered references in the main text.

Although the first documented collections were made by C.G. Pringle in 1885 and Edward Palmer in 1908 in the hills surrounding Chihuahua City as cited by Britton and Rose, the species was formally identified in its original description in 1923 as *Escobaria chihuahuensis* Britton & Rose (Figure 1) [9]. This classification depended on specific diagnostic features: unlike true *Coryphantha* (Engelm.) Lem. (which have seeds with reticulate testa and large flowers of central origin), *Escobaria* was characterized by having foveolate (pitted) seeds, smaller flowers, and fruits that keep persistent perianth remnants [9].

This distinction was not universally accepted. In 1929, Berger argued that these differences in seed and floral morphology did not justify generic segregation; consequently, *Escobaria* was downgraded to the rank of section or subgenus, and the species was transferred to *Coryphantha chihuahuensis* (Britton & Rose) A. Berguer [10]. This action ignited a decades-long debate. While some treatments restored *Escobaria* to full generic status in 1951 [14], others maintained its subordination to *Coryphantha*, citing transitional species [15]. The historical fluctuation between these genera is detailed in the timeline in Figure 1.

The discovery of population variants further complicated the discussion. *Escobaria henricksonii* Glass & R.A. Foster was described in 1977 based on populations that formed large caespitose clumps (in contrast to the mostly solitary habit of typical *chihuahuensis*) [16]. However, in 1998, this taxon was later downgraded to a subspecies (*Escobaria chihuahuensis* subsp. *henricksonii* Glass & R.A. Foster), as the differing habit was considered a variable trait rather than a clear barrier to speciation [17].

Towards the end of the 20th century, taxonomy sought new solutions. A proposal was made in 1999 to create the genus *Escocoryphantha* Doweld, intended to group “problematic” species sharing intermediate traits (Figure 1), although this classification did not gain widespread acceptance [11]. Finally, modern research on seed morphology has reinforced the original separation, demonstrating that seed testa microstructure (foveolate in *Escobaria* vs. reticulate in *Coryphantha*) is an evolutionarily stable and reliable trait for distinguishing the two lineages [18].

### 2.2. The Molecular Era: Phylogenetic Resolution and Transfer to Pelecyphora (2022)

A comprehensive phylogenetic study of the tribe Cacteae was conducted in 2022 [12], utilizing morphological data combined with five plastid markers (*matK*, *rbcL*, *psbA-trnH*, *rpl16*, *trnL-F*) targeting conserved plastidial regions [19,20]. This selection aligns with the standard proposed by the Consortium for the Barcode of Life (CBOL) Plant Working Group, which identified *rbcL* and *matK* as the core barcode for land plants [21]. These analyses indicated that the genus *Coryphantha* (in its broad sense) is polyphyletic and does not constitute a natural group. Instead, a strongly supported monophyletic lineage—the ‘*Pelecyphora*’ clade—was identified, comprising all *Escobaria* species, *Coryphantha macromeris* (Engelm.) Britton & Rose, and the classic *Pelecyphora* C. Ehrenb species (*Pelecyphora aselliformis* C. Ehrenb and *Pelecyphora strobiliformis* (Werderm.) Frič & Schelle ex Kreuz). Consequently, adherence to the principle of nomenclatural priority is required; since this genetically coherent group includes the type species of *Pelecyphora* (described by Ehrenberg in 1843), which predates *Escobaria* (1923) by 80 years, the International Code of Nomenclature mandates the use of the oldest valid name [22].

While the use of plastid markers was considered sufficient in that study to support the reclassification, nuclear ribosomal regions—specifically the Internal Transcribed Spacer (ITS)—emerge as more robust tools for species discrimination due to the significant phylogenetic signal found in these markers and their secondary structures [23]; for instance, the ITS2 region has demonstrated a discrimination rate of up to 92.31% in phylogenetic reconstructions, effectively outperforming standalone plastid markers such as *rbcL* and *matK*. Despite this utility, the application of ITS2 in automated identification (BLAST) remains limited by the scarcity of reference sequences in public databases. Therefore, the generation of ITS data for *P. chihuahuensis* represents a critical gap that future research must address to further refine its systematic placement.

Consequently, the authors formally proposed the combination *Pelecyphora chihuahuensis* (Britton & Rose) Aquino & Dan. Sánchez [12]. This reclassification is not just a label change but an acknowledgment that this species belongs to a distinct evolutionary lineage, sister to *Coryphantha* but separated from it millions of years ago.

To illustrate the complex nomenclatural history resulting from these revisions, Figure 1 provides a chronological overview of the generic re-classifications. It highlights the historical shifts from *Mammillaria* to *Coryphantha*, the creation of *Escobaria*, and the final placement in *Pelecyphora*.

Current taxonomy recognizes two infraspecific taxa: the typical subspecies (*subsp. chihuahuensis*) and subsp. *henricksonii* (formerly *Escobaria henricksonii*, as proposed by Taylor in 1998) [24,25].

## 3. *In Situ* and *Ex Situ* Conservation Approaches

Mexico is considered one of the most important regions for diversity and endemism of the Cactaceae family in the world. The country contains more than 70 percent of its cactus species as endemic plants, which grow in dry and semi-dry areas that face environmental degradation, land-use change, illegal exploitation, and climate change [26]. Given this vulnerability, both *in situ* and *ex situ* conservation methods are important to ensure the long-term survival of these species.

### 3.1. In Situ Methods for Cactus Conservation

**Figure 2:**
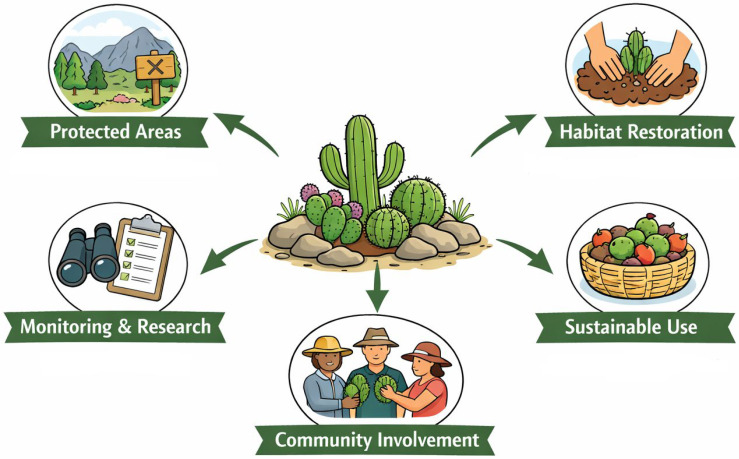
Overview of common *in situ* conservation methods used for cacti.

*In situ* conservation involves protecting cactus populations within their natural habitat by creating or strengthening protected natural areas, habitat restoration, monitoring and research, designing sustainable use, and including local communities in the management and preservation of the ecological processes that sustain their life cycles (Figure 2).

A summary of key *in situ* conservation methods is presented below:

#### 3.1.1. Protected Areas

According to Schwertner-Charão et al. [27], the establishment and effective management of protected natural areas, as well as strong laws that prevent the exploitation and illegal trafficking of wild cacti, are fundamental to preserving wild populations in their original habitats.

Establishing protected areas is a cornerstone of *in situ* conservation. The National Commission of Natural Protected Areas (CONANP) in Mexico manages 203 protected zone categories, which extend across approximately one million square kilometers of territory [28]. The protected zones fail to preserve many endangered cacti species because these plants exist only in specific areas, which include endemic species that have a limited geographic range [29]. The existing protected areas need to be reestablished according to the currently identified biodiversity conservation needs. Furthermore, these areas face operational challenges because illegal logging activities and land-use disputes and weak enforcement practices continue to exist even after two decades of their establishment [30].

#### 3.1.2. Habitat Restoration

Habitat restoration plays a vital role in reestablishing cactus populations that exist in areas where natural habitats have been degraded or completely destroyed. This process requires three specific tasks, which include native species planting, soil condition restoration and invasive plant removal. The restoration work becomes vital for arid regions because cacti require particular microhabitats together with nurse plants to survive [4]. In Arizona, for example, the *Echinocereus arizonicus* populations experienced successful restoration through the implementation of protective measures after their numbers declined due to mining and overexploitation activities [31].

#### 3.1.3. Monitoring and Research

Scientific monitoring is indispensable in tracking population dynamics, habitat conditions, and emerging threats. Long-term data allow conservationists to assess the effectiveness of interventions and adapt strategies accordingly [32].

A few assessments from the last fifteen years reveal that the conservation needs for species of the genus *Pelecyphora* are considerable on the grounds of their restricted populations and inadequacies in terms of available protected areas. Hernández and Gómez-Hinostrosa [5] pointed out that micro-endemic cacti such as *P. aselliformis* and *P. strobiliformis* often fall outside adequately devised conservation strategies, suggesting a need for targeted *in situ* monitoring. In concurrence, Badalamenti et al. [33] stressed that wild *Pelecyphora* populations are considered highly vulnerable. The document went on to examine propagation options that could cause a reduction in pressure on the natural populations. Moreover, Project CONABIO XA008 [34] stressed the absence of full-fledged field surveys and conservation status changes for *Pelecyphora*, sparking debates on major missing links that stand in the way of effecting success of *in situ* conservation programs. Here, all sources indicate the overwhelming requirements for monitoring, better protective actions, and an integrated strategy where the conservation needs for this highly threatened genus are to be met most effectively.

#### 3.1.4. Sustainable Use and Community Involvement

Integrating local communities into conservation initiatives ensures sustainability and reduces pressure on wild populations. Strategies include promoting legal cultivation, community nurseries, and education programs. Despite a limited number of previous publications examining sustainable use of *Pelecyphora* for its conservation, the broader conservation literature argues that, in essence, sustainable management schemes that accompany *in situ* protection provide successful conservation measures for endangered cacti. This view of Hernandez and Gómez-Hinostrosa [5] exemplifies the prospects of narrow endemic taxa like *P. aselliformis* and *P. strobiliformis* living in mostly unprotected areas, implying that habitat protection views alone are not sufficient and sustainable uses should be planned in line with strict conservation priorities. Navarro and Contreras-MacBeath [35] have placed significant emphasis on sustainable use strategies, seed management, and the cultivation of legal products of a sort that could overwhelmingly add value to conservation gains through community action. McGough [36] extends the scope of plant trade management by providing guidance on sustainable use options for high-value succulent species. Such policies are of critical interest for Pelecyphora taxa, which are currently facing significant pressure from international trade [4]. There is a lack of information regarding community participation and sustainable use of species of the genus *Pelecyphora*, indicating a significant knowledge gap [37]. However, several available case studies on other cacti in Mexico offer relevant analogs to guide the development of community-based conservation strategies applicable to species of this genus. In the Barranca de Metztitlán Biosphere Reserve, community-run nurseries have successfully shifted the focus from extraction to propagation, generating economic benefits while conserving biodiversity [38]. Similarly, in the Tehuacán Valley, traditional knowledge supports sustainable harvesting of columnar cacti, demonstrating how cultural practices can align with conservation goals [39]. These examples demonstrate the real ways in which communities are brought into the fold to help in the conservation of *Pelecyphora* in the wild, in situations where such approaches are yet to be implemented.

### 3.2. Ex Situ Methods for Cactus Conservation

**Figure 3:**
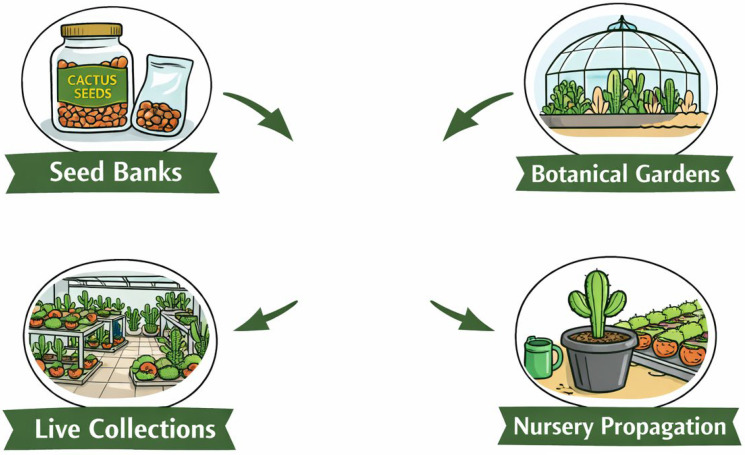
Overview of conventional *ex situ* conservation methods used for cacti.

*Ex situ* conservation acts as an essential complement when *in situ* protection alone is insufficient, particularly when populations are small, fragmented, or when there is imminent risk from human actions or natural disasters [40]. Some of the conventional methods include: (1) seed banks; (2) the creation of living collections in botanical gardens; (3) live collections; and (4) nursery propagation (Figure 3).

#### 3.2.1. Seed Banks

At present, no information is available in the literature concerning *ex situ* seed banking that is exclusive to the genus *Pelecyphora*, which points out the urgency of this situation in the conservation biology of this already limited and endangered group. However, studies on cacti that are either closely related or ecologically similar provide directions that are quite valuable for the seed bank techniques that can be used for *Pelecyphora*. In Brazil, cold storage extended seed viability in multiple cactus species [41]. Studies on *Lophophora diffusa* (Croizat) Bravo and genera like *Opuntia* Mill. and *Mammillaria* Haw. reveal that factors such as seed age, dormancy, and germination behavior are critical for successful storage [42,43]. These findings underscore the need for species-specific protocols in seed conservation.

#### 3.2.2. Botanical Gardens and Live Collections

Botanical gardens and living collections are very important for the *ex situ* conservation of cacti as they hold the genetic diversity and are the main places for research, public outreach, and long-term preservation. Institutions such as the Rio de Janeiro Botanical Garden and the Guimarães Duque Cactarium (CAGD) in Brazil hold extensive collections of endangered cacti, offering insights into species ecology, supporting education, and aiding conservation planning [44,45]. The CAGD, for example, maintains over 1000 specimens from 123 species, many of which are endemic or threatened [45]. Together, these cases highlight how botanical gardens can be a major player in creating strong *ex situ* strategies that might be used for the less prominent genus like *Pelecyphora*.

#### 3.2.3. Nursery Propagation

Nursery propagation secures plant material for restoration, research, and public distribution. For example, *Rhipsalis baccifera* (J.S.Muell.) Stearn achieved 100% rooting under optimized shade and soil conditions [45], revealing the significance of systematized environmental control for the success of propagation. Similarly, significant early growth was shown by *Haageocereus acranthus* (Vaupel) Backeb. 1933 under the controlled environment [46], which supported its role in use for restoration. Not only does the propagation of nursery plants play an essential role in the recovery of species, but it also contributes to community-based conservation through legal cultivation and trade. Altogether, these studies make a compelling case for in vivo propagation, which naturally enjoys a pivotal role in *ex situ* conservation by providing genetically diversified, well-acclimated plant materials that equally bolster both botanical gardens’ living collections and field restoration projects; this could be a framework that could be adapted for understudied genera such as *Pelecyphora*.

#### 3.2.4. Integration of *In Situ* and *Ex Situ* Conservation

One of the most useful remedies for long-term conservation, specifically of slower-growing and lower-reproduction species like many cacti, is to blend *in situ* and *ex situ* means [47]. Public policy could foster such types of connections by mandating biodiversity in sustainable development alongside agreements on international trade, such as CITES. This would keep collections under possession, unfold illegal collections and strengthen native species by ensuring their survival against threats of habitat destruction, overexploitation, and climate change [48].

Therefore, conservation of cacti in Mexico demands a multifaceted strategy on habitat protection, technological innovation, and active participation of the population to ensure their resilience and perpetuity in the face of daunting challenges faced by these iconic and ecologically important species.

## 4. Biotechnological Innovations in *Ex Situ* Conservation

Cacti, like many ornamental plant species, can reproduce through both sexual reproduction and vegetative propagation methods. The traditional propagation techniques that researchers use to propagate plants face multiple restrictions. The process of seed propagation that occurs naturally in the wild faces three main obstacles, which include low seed availability, poor germination rates, and the need for specific pre-germination treatments, which depend on the genus, with *Mammillaria* and *Echinocactus* species serving as examples [49,50]. The process of vegetative propagation through cuttings and grafting enables preservation of desirable phenotypes, which include color mutations that would otherwise disappear through sexual reproduction. The methods require extensive work because they need a high amount of time, and they have the potential to spread viruses [51,52]. The process of grafting one hectare of cactus plants requires 17,000 labor hours, which equals almost two years of nonstop work according to Jeong et al. [53]. The process of vegetative propagation becomes ineffective because certain cactus species lack the ability to create lateral branches and adventitious roots. The development of plant tissue culture methods provides scientists with effective propagation techniques that enable them to grow plants in large quantities while maintaining genetic diversity and protecting endangered cactus species that grow slowly.

Biotechnological innovations in conservation represent the transformative revolution in the preservation of high-concern species. This is particularly true for cacti genera. In this context, conservation strategies like *in vitro* propagation [54] and cryopreservation have the potential to provide leverage for gene pooling and consequent clonal multiplication [55,56]. Such techniques are geared to make the mass propagation and long-term preservation of rare species possible, while at the same time obtaining a better view of their molecular responses to stress and adaptation. In addition, methods such as CRISPR-Cas genome editing and synthetic biology would provide the greatest chance for enhancing resilience, adaptability, and genetic rescue for taxa at great risk [57].

Currently, no studies have been published in the literature discussing biotechnological methods regarding *P. chihuahuensis*. However, the acquired knowledge from other species of the family and even from the genus *Pelecyphora* can help formulate an *in vitro* propagation and conservation protocol, which constitutes a significant input to prevent the extinction of this species.

### 4.1. In Vitro Micropropagation

Cactaceae species face natural reproductive challenges due to low seed availability and low germination percentages, often requiring strict light and temperature conditions. Germination is typically slow or requires specific triggers for the few seeds to germinate successfully. *In vitro* propagation offers a biotechnological solution to overcome these barriers, enabling mass production of explants with desirable traits in limited space [54]. Consequently, Giusti et al. [58] utilized axillary shoots of *Pelecyphora aselliformis* Ehrenberg as plant material to examine the practicability of the micropropagation technique, using Murashige and Skoog’s [59] medium supplemented with growth regulators like Thidiazuron (TDZ), 2,4-Dichlorophenoxyacetic acid (2,4-D), kinetin (Kin), and 6-Benzylaminopurine (6BAP). The advantageous effect of TDZ was that it led to callus formation and eventually to the production of plantlets. In contrast, with Kin, there was a corresponding high percentage of shoots that were multiplied and proliferated along with good quality, but calluses were either not induced or were in scanty amounts. In another research, areoles derived from the two endangered cacti of Mexico (*P. aselliformis* and *P. strobiliformis* Werdermann) were grown on MS medium, after 60 days of incubation with 8.8 µM 6BAP and 30 g L^−1^ of sucrose. *P. aselliformis* gave 13.7 shoots per explant, while the same treatment with only 50 g L^−1^ of sucrose for *P. strobiliformis* produced 12.4 (shoots per explant). The first explants underwent three subcultures and were elongated by adding 3 g L^−1^ of activated carbon to the culture medium, followed by root formation obtained with 2.85 µM indoleacetic acid (IAA), 5.71 µM of IAA, 2.46 µM indolebutyric acid (IBA), or 4.90 µM of IBA. During this phase, there was no significant difference among the plant growth regulators applied, 89% of the explants rooted for *P. aselliformis* and 87% of *P. strobiliformis*. In the end, the survival rate of the seedlings in acclimatization phase was 88% after 16 weeks when using sand and soil (1:1) as substrate [60].

As described by Arias [61], cytokinins such as Kin and 2iP, when added to the culture medium, stimulated phylloclade formation to a greater extent than 6BAP in *P. strobiliformis*. Another study instated the use of seeds of *P. aselliformis* species as initial plant material and a subculture of longitudinal explants in the MS medium supplemented with 8.8 μM benzyladenine (BA) or 4.6 μM kinetin. The number of such subcultures increased to five shoots per explant, up to 25%. Superior plant material quality was obtained in the BA medium, but hyperhydricity (a physiological disorder distinguished by the glassy or translucent appearance of tissues of the plant due to overhydration) was comparable in both growing media [62].

Studies on the *in vitro* culture of the genus *Coryphantha* have been conducted by a number of researchers. According to Sánchez et al. [12] and Chincoya et al. [63], this genus is closely related to *Pelecyphora* in terms of phylogeny, which is why some of the micropropagation results are presented in Table 1.

A significant portion of the culture work on species of the genus *Coryphantha* in most of the indexed journals is at least ten years old; newer studies on these species are, however, a little harder to come by, despite the fact that many of them are threatened or endangered.

Although not addressed in detail in this review, the assessment of genetic fidelity in micropropagated plantlets is a key component of reliable *in vitro* conservation strategies, as somaclonal variation can compromise the genetic integrity of rare or endangered species [71].

### 4.2. Cryopreservation

Currently, as explained previously, due to the increasing number of endangered or threatened species, whether from habitat loss or climate change, the need for the process of *ex situ* conservation is recognized to be of help. Cryopreservation is one method that preserves explants or tissues at ultra-low temperatures and potentially long-term. Liquid nitrogen is most used for these purposes [55].

Quatrano [72] was the first to cryopreserve plant cells of *Linum usitatissimum* L. by cryoprotectant (dimethyl sulfoxide-DMSO). It can be carried out for cells with low water level (e.g., seed, pollen) by the use of liquid nitrogen (−196 °C), but when the water content is high, the breaking of tissues will occur due to the formation of ice crystals. Hence the application of slow cooling with cryoprotectants seems necessary for exploring this methodology for more species of plants [66]. Regarding the Cactaceae family, only four articles have addressed this topic since 2010, and they focus on endemic species from Brazil.

In an early study, Veiga-Barbosa et al. [73] investigated cryopreservation in seven different cactus species (*Discocactus zehntneri* Britton & Rose, 1922, *Melocactus concinnus* Buining & Brederoo, *Melocactus albicephalus* Buining & Brederoo, *Melocactus paucispinus* Heimen & R.J. Paul, *Pilosocereus gounelli* (F.A.C.Weber) Byles & G.D.Rowley, *Cereus jamacaru* (DC.) Kostel., 1835, and *Micranthocereus flaviflorus* Buining y Brederoo 1974), which were plunged directly into liquid nitrogen for different intervals (7, 30, or 120 days). The findings revealed that cryopreservation had no impact on the percentage of germination, and the only species that exhibited a decline in seed germination after 120 days of preservation in liquid nitrogen was *M. albicephalus*, where the seeds went from 84 to 88% (7 and 30 days, respectively) to 54% (120 days).

Expanding on this work, Marchi et al. [74] examined the response of seeds from three species (*Stephanocereus luetzelburgii* (Vaupel) N.P. Taylor & Eggli, *Pilosocereus gounellei*, (F.A.C.Weber) Byles & G.D.Rowley and *Discocactus zehntneri* Britton & Rose, 1922) native to Bahia, Brazil, were tested for 0, 7, and 30 days in liquid nitrogen. Results showed that the germination rate of the three species was not adversely affected by exposure to liquid nitrogen. Interestingly, the highest values were obtained with the species of *D. zehntneri*, with 20% germination after 30 days of cryopreservation compared with 0% in the control and 3% after 7 days at −196 °C.

Further studies by Civatti et al. [75] assessed cryopreservation in three additional species native to Bahia-*Melocactus conoideus* Buining & Brederoo, *Micranthocereus polyanthus* (Werderm.) Backeb, and *M. flaviflorus*. Seeds were immersed in liquid nitrogen for either 7 or 30 days, while the control seeds were sown the same day without being cryopreserved. After freezing, the seeds were placed on half-strength MS medium with agar and sucrose, and germination was checked every day for 21 days. *M. conoideus* seeds did not differ significantly in germination between the control group and those cryopreserved for 7 days. On the contrary, after 30 days of cryopreservation, the seeds did not germinate even though 84.6% of them were still alive. A similar trend was seen in the other two species; the control and 7 days of cryopreservation recorded the highest germination percentages, and there were no significant differences between these treatments (35.0 and 58.8% for *M. flaviflorus*; 18.8 and 22.5% for *M. polyanthus*). On the other hand, 30 days of cryopreservation resulted in a sharp reduction in germination, with *M. flaviflorus* and *M. polyanthus* having 16.3% and 11.2%, respectively.

Most recently, Vendrame et al. [56] evaluated the cryopreservation potential of *Melocactus zehntneri* Braun ex Ritter f. and *Cereus gounellei* Luetzelb ex Schum k. seeds subjected to 10 days of exposure to liquid nitrogen (–196 °C) under various treatment conditions, including the use of cryoprotectants such as PVS2, PVS2 supplemented with 1% phloroglucinol, 1% Supercool, or a combination of both additives. According to the method of Sakai et al. [76], the PVS2 cryopreservation solution is made of 30% (*w*/*v*) glycerol, 15% (*w*/*v*) dimethyl sulfoxide (DMSO), 0.4M sucrose and 15% (*w*/*v*) ethylene glycol. After the cryopreservation period, the seeds were allowed to germinate for 15 days. All treatments, including the control (where no cryoprotectants were used), showed germination rates between 77% and 89% for *M. zehntneri* and between 79% and 95% for *C. gounellei*. Healthy seedlings without abnormalities were observed in both species. The authors concluded that cryoprotectants are not necessary for these two species; the seeds can be cryopreserved directly in liquid nitrogen.

Taken together, these studies demonstrate the potential for seed cryopreservation as a viable strategy for the long-term conservation of Cactaceae germplasm, although species-specific responses and optimal storage durations must be carefully considered.

### 4.3. Genomic and Transcriptomic Tools as a Framework for Cactus Conservation

Genomic tools serve as vital instruments that help scientists protect cacti (Cactaceae) through their ability to reveal genetic diversity and evolutionary relationships and physiological adaptation patterns. The molecular study of cactus physiology and stress resilience remains in its initial stages, yet recent genomic research has produced essential findings that will enable future developments.

The assembly tools SPAdes and Canu have played essential roles in mapping out the complete genetic material of cactus genomes. The researchers used SPAdes, optimized for short-read Illumina data, to build the entire mitochondrial genome of *Mammillaria huitzilopochtli* [77]. In contrast, Canu successfully produced accurate long-read assemblies, which included *Opuntia basilaris* Engelm. & Bigelow [78] and *Carnegiea gigantea* (Engelm.) Britton & Rose [79] as test cases. The Cactaceae genetic material, which scientists obtained through these assemblies, now serves as a basic foundation for their upcoming research on genetic material and evolutionary patterns.

With genomic data ready to use, strategies for gene prediction and structure annotation, e.g., the AUGUSTUS and MAKER annotation platforms, are commonly adopted. For example, Armstrong et al. [80] used Progressive Cactus for the purpose of creating whole-genome alignments. HAL files were made into fast MAF files for downstream analyses using a script (*hal2maf_split.pl*) included with AUGUSTUS and the MAF file. Here, well-annotated reference genomes were also appropriately applied to ensure gene structure integrity. Aside from this, MAKER has also been of paramount importance to comment on nuclear genomes of species like *O. basilaris* [78], *C. gigantea* [79], and *Cereus fernambucensis* Lem., 1839 [81].

Transcriptomic approaches have further accelerated gene discovery in non-model plants, including cacti. Differential gene expression analysis, in particular, identifies genes that respond to abiotic stresses such as drought, salinity, and temperature, revealing adaptation mechanisms at a molecular level. For example, *Mammillaria bombycina* Quehl was studied under *in vitro* dehydration conditions, generating over 84,000 transcripts through Illumina sequencing, with the expression of glyoxalase pathway genes validated by qRT-PCR [82]. Similarly, *Melocactus glaucescens* Buining & Brederoo—an endangered ornamental cactus—was transcriptomically profiled for *in vitro* shoot induction. Researchers identified over 2000 differentially expressed unigenes, including upregulation of stress-related genes like WIND1 and CALMODULIN [83]. These transcriptomic resources are critical for understanding propagation and stress responses in cacti.

Genomic markers, particularly SSRs (simple sequence repeats), also play a vital role in characterizing genetic diversity. More especially, MISA has been applied in the identification of SSRs in the chloroplast and mitochondrial genomes of *Opuntia cochenillifera* (L.) Mill. for cultivar classification and population structure analysis [84]. Bioinformatic tools like OrthoFinder allow for the identification of orthologous gene sequences and the definition of ‘orthogroups,’ providing biologists with a better understanding of adaptive divergence in cacti [85].

**Figure 4:**
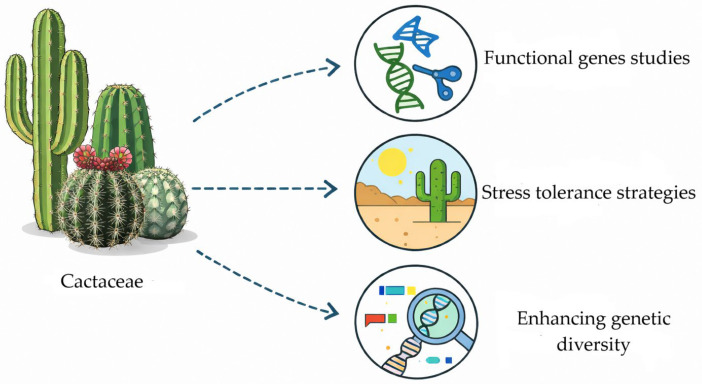
Proposed conceptual framework for the application of CRISPR/Cas genome editing in the conservation of Cactaceae.

CRISPR/Cas technology is revolutionizing plant biotechnology by enabling precise and heritable gene editing. While its application is well established in model and crop plants, its potential for conserving endangered and slow-growing taxa like cacti remains untapped. Species such as *P. chihuahuensis* face growing threats from habitat loss and climate change, making CRISPR/Cas a promising tool for exploring gene function, enhancing stress tolerance, and restoring adaptive traits. Although no studies have yet applied this technology to cacti, its future use in the family is promising. Figure 4 presents a conceptual framework illustrating potential applications of CRISPR/Cas in cactus conservation, including gene function analysis, development of resilient phenotypes, and enhancement of genetic diversity.

The best-known application of CRISPR/Cas technology is low-cost, easy-to-use genome editing. Regarding species conservation, CRISPR/Cas offers theoretical pathways for genetic rescue or the introduction of adaptive alleles into endangered species [57].

The most significant possible uses of this technology include saving genes from species that face extinction and protection of endangered species. The particular species experience diminished genetic variation because their habitats face destruction and their members breed within their own population. CRISPR technology enables researchers to fix genetic defects, which will also increase the available genetic resources for research [86]. Another important application involves the use of CRISPR to create disease-resistant organisms because they can use the technology to change essential resistance genes [57,87]. The technology has seen increased usage because plant diseases cause both lower crop production and reduced fruit quality, which requires better solutions. Additionally, CRISPR may be used to restore extinct traits by creating new genetic material, which will bring back traits that vanished after the species went extinct [88].

The knowledge of the genomes of the Cactaceae family, threatened or endangered species, will dictate the application of this technology, as the gene editing precision will be reliant on the genome assemblies’ quality [89]. To date, fewer than ten cactus genomes have been published, highlighting the limited genomic resources available for this plant family. Some of the presently accessible genome assemblies are presented in Table 2, drawing attention to the considerable work to be done to propel genomic research and aid the conservation of these species that are ecologically and culturally important.

The complementary use of modern genetic tools and gene editing technologies like CRISPR/Cas has formed a comprehensive framework for the consociation of cacti. While application of CRISPR/Cas in cacti remains largely prospective, it has strong potential for enhancing stress tolerance and preserving key adaptive genetic traits. Both of them act hence on *in situ* and *ex situ* conservation by laying a firm molecular ground for taxonomic resolution, environmental adaptation understanding, safekeeping and utilization of genetic resources.

Significant in the lack of current genomic and transcriptomic data on *P. chihuahuensis* or other species within the genus *Pelecyphora*, the continually growing body of science among related species in Cactaceae serves as a more relevant basis of reference for these explorations. Significant improvements have thus been made with respect to genome science and the like in underexamined and imperiled taxa, thereby supporting evidence-based conservation and restoration initiatives.

## 5. Biocultural and Policy Perspectives

The relationship between humans and cacti on the American continent, especially in Mexico, is so ancient and profound that it is part of the national coat of arms [96]. Beyond their ecological role, numerous species have been vital economic and cultural resources, serving as sources of food, medicine, and ceremonial materials [3]. However, most species currently face complex socioeconomic and regulatory pressures, putting endemic species like *P. chihuahuensis* at the heart of debates on their conservation and sustainable management.

### 5.1. The Biocultural Heritage of Cacti in the Chihuahuan Desert

Cacti are a cornerstone of Mexico’s biocultural heritage. Since pre-Hispanic times, these plants have been connected to the development of desert cultures, providing essential resources. The stems (nopalitos) and fruits (tunas, pitayas, xoconostles) of different species have served as a key food source, eaten fresh or processed into products like jams, vinegars, and fermented drinks [97,98]. Traditional medicine in Mexico and other parts of the Americas uses many cactus species to treat various ailments: diabetes, wounds, burns, digestive issues (gastritis, ulcers), kidney problems, fever, respiratory illnesses, heart issues, rheumatism, among others [98,99]. Pharmacological research has started confirming some of these uses by identifying bioactive compounds. For example, steroids with antidiabetic properties have been found in the roots of *Peniocereus greggii* (Engelm.) Britton & Rose [99], and extracts from various Mexican medicinal plants, including cacti, have shown significant antibacterial activity [100]. The mucilages found in various species offer soothing and mucosal protection benefits [97,101]. Additionally, species like *Lophophora williamsii* (Lem. ex J.F.Cels) J.M.Coult (peyote), which is endemic to this region, hold deep ceremonial and spiritual importance that persists in the rituals of indigenous peoples today [102]. Nopales (genus *Opuntia*) are the natural habitat of the cochineal insect (*Dactylopius coccus*). This insect is the source of carminic acid, the precursor of carmine, a naturally occurring red pigment with an intense color. This dye has been and remains highly valued in the food, cosmetics, and textile industries throughout history [97]. *Opuntia* cladodes, especially those of *Opuntia ficus-indica* (L.) Mill, are an essential source of fodder for livestock (cattle, goats, sheep) in many arid and semi-arid regions of the world, particularly during droughts. The spines typically must be burned or mechanically removed before the plants can be fed to livestock [97]. Historically, some columnar species, such as *Marginatocereus marginatus* (DC.) Backeb, formally known as *Pachycereus marginatus* (DC.) Britton & Rose (“organ”) have been used to create living fences due to their rapid growth and dense spines. They are planted in rows to delimit properties and corrals [103]. The dry, light wood of some large columnar cacti (“quiote”) has traditionally been used in rural construction for beams, roofs, and furniture [3].

In the modern era, this biocultural value has evolved. In addition to subsistence uses, there has been an intense global appreciation of its ornamental value, which has consolidated a trade in horticulture and collecting [13,26,104]. Species such as *P. chihuahuensis*, with their tiny size and intricate spine patterns, are highly prized for their beauty. This new facet of human–plant interaction, however, has generated a demand that fuels the illegal extraction of wild populations [104], paradoxically turning esthetic value into one of the greatest threats to their survival.

### 5.2. Illegal Trafficking and Socioeconomic Pressure

Appreciation for cacti has transcended local borders, giving rise to a robust international market of collectors willing to pay high prices for rare specimens with unique morphologies or restricted distribution [26,104,105]. This global demand is the primary driver of illegal trafficking, an activity that exerts devastating pressure on the natural populations of many species. The collection of live plants and seeds for the horticultural trade and for private collections is the main driver of extinction risk, affecting 47% of threatened cactus species [4,26,104]. Slow-growing and highly endemic species, such as *P. chihuahuensis*, are particularly vulnerable, as 86% of threatened cacti used in horticulture are taken directly from their habitats [4]. Selective extraction of the most attractive individuals not only reduces population size but also decreases their long-term viability by removing the most successful breeders.

This phenomenon creates a complex socioeconomic network. At the local level, illegal collection can represent a source of income for specific communities. In contrast, at the global level, it fuels a trade chain, often via the internet, that culminates in private collections in Europe, Asia, and North America [104]. Thus, the esthetic value that a collector assigns to a cactus in a distant market translates into a direct and tangible threat to the biodiversity of the Chihuahuan Desert [4,104].

### 5.3. Regulatory and Policy Framework for Conservation

Internationally, the species is listed in Appendix II of CITES to regulate its cross-border trade [106]. However, the effective enforcement of this regulation relies on the accurate identification of specimens at ports of entry. As highlighted by Yesson et al. [107], this process is often hindered because seized cacti are frequently sterile or fragmented, lacking the diagnostic morphological features (flowers or fruits) required for traditional classification. In this context, DNA barcoding serves as a critical forensic tool, enabling the rapid and accurate identification of regulated species to ensure compliance with CITES mandates.

However, beyond trade regulations, formal risk assessments present a contrasting scenario. The IUCN Red List classifies the species as “Least Concern” (LC), based on a 2009 assessment that reports its population as “stable” [108]. Similarly, Mexican legislation does not grant it specific protection. The species was not included in the Mexican Official Standard NOM-059-SEMARNAT-2010 [109] or in its 2025 Draft Amendment [110,111]. Inclusion in this standard is determined by the “Plant Extinction Risk Assessment Method” (MER-Plantas), a system based on criteria of distribution, habitat, vulnerability, and human impact (Table 3). The final assessment sums these values to assign specific risk categories—Endangered (P), Threatened (A), or Special Protection (Pr)—as detailed in the table criteria. Currently, the lack of robust data prevents *P. chihuahuensis* from reaching the score required for legal protection.

Table 3 summarizes the specific criteria and scoring methodology used to evaluate a species’ extinction risk for inclusion in NOM-059-SEMARNAT-2010, formally known as the Method for Evaluation of Risk of Extinction of Plants (MER-Plantas). This quantitative method generates a cumulative score derived from four independent indices: Rarity (A), Habitat Characteristics (B), Biological Vulnerability (C), and Anthropogenic Impact (D).

The final score is calculated as the sum of the subtotals of these four criteria. Based on the resulting value, a species is assigned to one of the specific risk categories defined by Mexican law: Endangered (En Peligro de Extinción, P): ≥2 points; threatened (*Amenazada*, A): >1.7 and <2 points; subject to Special Protection (Sujeta a Protección Especial, Pr): ≥1.5 and <1.7 points.

### 5.4. Integrating Scientific Evidence, Public Policy, and Social Action

The effective conservation of *P. chihuahuensis* is currently at a crossroads, defined by a notable regulatory discrepancy. Although the species is listed in Appendix II of CITES, which regulates its international trade, it is legally vulnerable within its own territory because it is absent from the primary national protection tool [111].

This regulatory gap is not merely an administrative issue; it is symptomatic of a lack of specific scientific information. This same data deficiency likely explains why the IUCN assessment has not been updated since 2009 [108]. In fact, the current entry is explicitly annotated as ‘Needs updating’ by the IUCN itself, leaving the species listed as ‘Least Concern’ despite the increasing habitat fragmentation and extraction pressures inferred from regional trends [4,5].

As detailed in the MER-Plantas methodology (Table 3), inclusion in NOM-059-SEMARNAT-2010 requires robust quantitative data regarding geographic distribution, habitat specificity, and biological vulnerability. Historically, the lack of such detailed information has hindered formal assessments to justify the legal protection of *P. chihuahuensis*. This is precisely where biotechnological tools act as an indispensable bridge. Scientific evidence generated by molecular phylogenetics and species distribution models provides the complex, up-to-date data required to satisfy the MER-Plantas criteria. Translating this evidence into “public policy” is the next logical step. With this data, as has been done for other species [110], it is possible to formally request the reevaluation of *P. chihuahuensis* for inclusion in NOM-059. Such inclusion would provide a solid legal framework for *in situ* protection, justifying management plans and strengthening penalties for illegal extraction.

Simultaneously, biotechnology activates “social action” through *ex situ* strategies. While legal protection is being consolidated, *in vitro* micropropagation and cryopreservation offer viable methods for mass propagation and germplasm preservation [112,113]. Developing these protocols enables sustainable cultivation programs involving botanical gardens, nurseries, and collectors. This approach seeks to meet ornamental demand with plants of legal origin, thereby reducing poaching pressure on wild populations [13]. Taken together, this integration of evidence-based science, informed public policy, and participatory social action represents a holistic strategy to ensure the long-term survival of this Chihuahuan Desert endemic.

Finally, conservation efforts necessitate strict adherence to ethical standards. Given the pressures of illegal trafficking, it is imperative that any academic activity involving fieldwork or sampling operates under specific Scientific Collection Permits issued by the relevant national authority (SEMARNAT). These permits ensure that research does not compromise wild populations and align with international frameworks such as the Nagoya Protocol on Access to Genetic Resources [114]. Compliance with these ethical guidelines safeguards sovereign rights over biodiversity and strictly distinguishes legitimate scientific inquiry from unauthorized extraction.

## 6. Conclusions

*Pelecyphora chihuahuensis* is one of the least known Mexican indigenous cacti, with increasing time constraints due to habitat destruction, climate change, and illegal activities. The particularity of the species is immensely troubling, with no sources of data at the taxonomic, ecological, or conservation levels. There is mounting urgency for specific studies. Integrating molecular techniques, distribution modeling, and conservation biotechniques seems to present a tidy way out to fill the existing knowledge holes and feed into evidence-based conservation strategies. While advanced approaches such as *in vitro* culture, gene or gene function applications, or cryopreservation help in generating a number of valuable troops, their application on *P. chihuahuaensis* will depend on their species specificity and accountable ethical and ecological issues. In essence, a multidisciplinary framework should offer a bridge between taxonomy, ecology, and biotechnology forms for the long-term conservation of studying and managing *P. chihuahuensis*, as well as to advise on conservation policy for threatened and endangered autochthonous cacti in arid and semi-arid environments.

## 7. Research Gaps and Future Directions

The desert-dwelling cactus *P. chihuahuensis* remains one of the least researched endemic cacti in northern Mexico, in view of its ecological, evolutionary, and conservation significance. Most of what is known is fragmentary and very often inferred from similar taxa, reflecting huge gaps within the taxonomic, ecological and biotechnological domains.

One of the gaping holes is that there are no comprehensive molecular and population-level genetic studies of the species *P. chihuahuensis*. As there is no species-specific genomic dataset available so far, this has resulted in scanty genetic diversity estimates, weak population structure, and poor evolutionary history. Future research should put principal emphasis on developing molecular markers and genome-wide studies by making use of next-generation sequencing methods to help elucidate the intraspecific variation and resolve taxonomic confusion within the *Pelecyphora* genus. Such data serve as the foundation for identifying units for conservation and serve as the basis for both *in situ* and *ex situ* programs.

Biotechnological resources have been recognized as invaluable for cactus preservation for quite a while now, but their application to *P. chihuahuensis* is still largely theoretical. There are currently no standardized protocols for *in vitro* propagation, somatic embryogenesis, or cryopreservation of this species. Future work should be devoted to developing species-specific techniques of tissue culture and cryostorage in order to alleviate pressure from illegal collection and support *ex situ* conservation efforts. Also, physiological and genetic stability of regenerants must be thoroughly checked before any massive scaling.

Sophisticated technologies, like CRISPR-Cas systems and synthetic biology, bring out new, promising techniques for the conservation of the rare cacti. However, the application for *P. chihuahuensis* is still hypothetical, as no practical experiments have been conducted to determine its likelihood, risk, or ethical consequences. A vibrant and dynamic discussion is needed to determine whether these technologies could be useful in the achievement of conservation missions while being cautious about genetic conservation and regulatory compliance.

Integrative research becomes crucial in embedding taxonomy, ecology, and biotechnology in a conservation-oriented context after all. That integration could present a very definitive mechanism toward evidence-based conservation policies by combining research of genomics with ecological modeling and bio-farming. Strengthened dialogue between researchers, conservation government bodies, and local communities will be imperative in communicating such advances to actionable management and protection of *P. chihuahuensis* on a long-term and sustainable scale.

## Figures and Tables

**Table 1:** *In vitro* propagation studies in the genus *Coryphantha*: explant sources, culture conditions, and main outcomes.

Species	Culture Medium and Growth Regulators	Main Results	References
*Coryphantha elephantidens* (Lem.)	MS with 1.5 mg L^−1^ 6BAP or Kin.	6.9 (transverse) and 12.4 (longitudinal) axillary shoots.	[64]
*Coryphantha retusa* (Britton & Rose)	Half-strength MS + 6BAP (0–3 mg L^−1^), NAA (0–1 mg L^−1^).	Highest bud propagation at 2 mg L^−1^ 6BAP; rooting in MS without hormones.	[65]
*C. elephantidens*	MS + 2,4-D (0.2–2.0 mg L^−1^), Kin (0.5–1.5 mg L^−1^), various antibiotics.	Callus induction; kanamycin enhanced morphogenesis.	[66]
*C. elephantidens*	MS + auxins (IAA, IBA, NAA, 2,4-D: up to 4.4 µM), cytokinins (up to 18.4 µM).	Max. shoot regeneration with 6.9 µM Kin + 2.3 µM 2,4-D; 100% survival after acclimatization.	[67]
*C. elephantidens*	MS + 4.6 µM Kin, auxins (2,4-D, IAA, NAA, IBA) at varied concentrations.	Enhanced callus and shoot induction with 9 µM 2,4-D + 4.6 µM Kin.	[68]
*Coryphantha radians* (DC.) Britton & Rose	MS + 1 mg L^−1^ BA, 0.5 mg L^−1^ IBA.	Avg. 4.15 ± 0.475 shoots per explant.	[69]
C. macromeris actually *Pelecyphora macromeris* (Engelm.) D.Aquino & Dan.Sánchez	MS + 44 µM BA, 0.5 µM 2,4-D, thiamine, sucrose, inositol.	Callus maintained for 4 years; up to 20 shoots per tube in 6–8 weeks.	[70]

MS—Murashige and Skoog medium; 6BAP—6-benzylaminopurine; NAA—α-naphthaleneacetic acid; 2,4-D—2,4-dichlorophenoxyacetic acid; Kin—Kinetin; IAA—Indole-3-Acetic Acid; IBA—Indole-3-butyric acid; BA—Benzyladenine.

**Table 2:** Presently available genome assemblies in Cactaceae family.

Species/Genome Resource	Genome Type	Notes
*O. basilaris*	Chromosome-level nuclear assembly (GenBank accession numbers: SRR30989399, SRR30989400)	It represents the first nuclear genome to be sequenced in subfamily Opuntioideae, and the most complete cactus nuclear genome at present [78]
*Opuntia* spp. and 32 Cactaceae’s species	Plastome analysis (sizes ranging from 121,985 bp to 152,717 bp). The GenBank accession numbers for the 16 species analyzed are available in Table 1 of the cited article.	As a result, 101 species-specific SSRs and six plastid markers were identified in Opuntia plastomes, offering effective tools for precise germplasm identification and molecular studies [90]
*Selenicereus megalanthus* (K. Schum. ex Vaupel) Moran	Chromosome-level nuclear assembly (The data are available in the NCBI Sequence Read Archive (SRA) under BioProject accession number PRJNA1117350.)	*S. megalanthus* is an auto-tetraploid with high heterozygosity (AAAB) [91]
*Selenicereus polyrhizus* (A.Berger) Britton & Rose, 1909	Chromosome-level nuclear assembly (The data are available at Genome Warehouse in National Genomics Data Center, Beijing Institute of Genomics, under accession numbers GWHEUSQ00000000.1 and GWHEUSR00000000.1)	The high-quality genome assembly of two haplotype [92]
*C. jamacaru*	Chromosome-level nuclear assembly (GenBank under the accession number JBOBQE000000000)	The assembly is highly complete and contiguous, with 1652 sequences totaling approximately 1.64 Gb [93]
*Opuntia ficus-indica* (L.) Mill., 1768 and *Opuntia robusta* H.L.Wendl. ex Pfeiff., 1837	Sequence and de novo assemble using nuclear genomic DNA (sizes ranging in length from 70 to 125 bp).	Although only a limited number of genomic sequences were collected, a significant portion could not be annotated in the NCBI NR or UniProtKB/SwissProt databases, as the Opuntia sequences primarily matched proteins from other plant species [94]
*C. gigantea*	Nuclear genome sequences were obtained for the progeny: SGP5p, with a length of 5,085,408 bp, and SGP5, with a length of 648,566 bp. This Whole Genome has been deposited at DDBJ/ENA/GenBank under the accession number JAKOGI000000000	Improved saguaro assembly [79]
*Mammillaria huitzilopochtli* D.R. Hunt, 1979	Mitochondrial genome with a linear chromosome length of 2,052,004 bp. The genome generated has been deposited at GenBank under the accession number OP081771	First mitochondrial genome (mtDNA) assembly for cactus [77]
*C. fernambucensis*	Nuclear genome. The datasets generated and/or analyzed during this study are available in the NCBI SRA database under project ID PRJNA587492	~1.58 Gb assembly with comparative data [81]
*Hylocereus undatus* (Haw.) D.R.Hunt, 2017	Chromosome-level nuclear assembly. The Whole Genome Shotgun accession number is JACYFF000000000	The chromosomal-level genome assembly contains 11 longest scaffolds corresponding to the 11 chromosomes [95]

**Table 3:** Method for evaluation of risk of extinction of plants in Mexico (MER-Plantas).

Index and Criterion	Subcriteria and Scoring	Maximum Score	Subtotal Calculation
I. RARITY INDEX			
A. Geographic Distribution	(1) Extent of distribution:	11	Subtotal A = (Sum A/11)
	≤1 km^2^ = 4		
	>1 km^2^ but <1% of National Territory = 3		
	>1–≤5% of National Territory = 2		
	>5–≤40% = 1		
	>40% = 0		
	(2) Number of populations:		
	1–3 = 3; 4–8 = 2; 9–25 = 1; ≥26 = 0		
	(3) Number of biogeographic provinces:		
	1 = 3; 2–3 = 2; 4–5 = 1; ≥6 = 0		
	(4) Representativeness of distribution:		
	Peripheral/Extralimital = 1; Non-peripheral = 0		
B. Habitat Characteristics	(1) Number of vegetation types present:	9	Subtotal B = (Sum B/9)
	1 = 3; 2 = 2; 3 = 1; ≥4 = 0		
	(2) Specialized habitat: Yes = 1; No = 0		
	(3) Dependence on primary habitat: Yes = 1; No = 0		
	(4) Dependence on disturbance: Yes = 1; No = 0		
	(5) Altitudinal range:		
	<200 m = 3; 200 m–<500 m = 2		
	500 m–<1000 m = 1; ≥1000 m = 0		
C. Biological Vulnerability	C1. Demography:	23	Subtotal C = (Sum C/23)
	(1) Total no. of individuals: ≤500 = 3; 501–5000 = 2; 5001–50,000 = 1; ≥50,001 = 0		
	(2) Recruitment: All populations = 0; Some populations = 2; Absence = 4		
	(3) Demographic attributes (Yes = 1, No = 0):		
	Density dependence		
	Clonality		
	Population decrease		
	High fecundity variance		
	Dioecy		
	Few propagules		
	Synchronous flowering		
	C-2. Genetics:		
	(1) Molecular variation: Low (10%) = 1; High (>10%) = 0		
	(2) Molecular genetic structure: Low (20%) = 1; High (>20%) = 0		
	(3) Amount of genetic variation: Low = 1; High = 0		
	(4) Differentiation level: Low = 0; High = 1		
	C-3. Specialized biotic interactions (Yes = 0, No = 1 [except as noted]):		
	(1) Nurse plant required?		
	(2) Specific host required?		
	(3) Specific pollinator required?		
	(4) Specific disperser required?		
	(5) Obligate myrmecophily?		
	(6) Is mycorrhizal dependence strict?		
	(7) Significant effect by predators/pathogens?		
II. ANTHROPOGENIC IMPACT INDEX			
D. Impact of Human Activity	(1) Habitat alteration: Benefited = −1; No effect/Unknown = 0; Harmed = 1	10	Subtotal D = (Sum D/10)
	(2) Impact level of human activities on habitat:		
	Precludes population viability = 4		
	Strong, affects all populations = 3		
	Strong in some/moderate in all = 2		
	Moderate, affects only some = 1		
	No significant impact = 0		
	(3) Deterioration due to global changes: No = 1; Yes = 0		
	(4) Impact of use (extraction):		
	Population removal = 4		
	Strong, affects all populations = 3		
	Strong in some/moderate in all = 2		
	Moderate, affects only some = 1		
	No impact = 0		
	(5) Cultivated or propagated *ex situ*: No = 1; Yes = 0		

## Data Availability

No new data were created or analyzed in this study. Data sharing is not applicable.
